# Gender-based violence and femicide interventions-perspectives from community members and activists in South Africa

**DOI:** 10.3389/fgwh.2024.1199743

**Published:** 2024-07-24

**Authors:** Sibusiso Mkwananzi, Motlalepule Nathane-Taulela

**Affiliations:** ^1^ Faculty of Health Sciences, University of the Witwatersrand, Johannesburg, South Africa; ^2^ Department of Social Work, University of the Witwatersrand, Johannesburg, South Africa

**Keywords:** gender-based violence and femicide, intervention, community members, South Africa, activists

## Abstract

Globally, Gender-Based Violence and Femicide (GBVF) remains a multifaceted social issue in the 21st century. Despite the ratification of international treaties and national laws, South Africa continues to have alarmingly high levels of GBVF, which were worsened during the Covid-19 national lockdown. The 2020 crime statistics reported that one in five South African women were victims of GBVF and South African Police Services (SAPS) data from 2015 to 2020 showed that seven women are killed daily nationwide. Despite copious studies on GBVF, the voices of local community members and activists as key collaborators in such research have been excluded. Therefore, this study used a mixed methods approach to determine the known interventions to decrease or eliminate GBVF and their effectiveness in seven communities across South Africa. The study included 191 participants in a survey for the quantitative aspect as well as a qualitative aspect of the study. Community members participated in gender-specific focus group discussions, while activists had a focus group of their own to obtain data on interventions. Our findings showed that three main forms of interventions existed in South Africa, viz. government-led campaigns that normally occurred during the 16 days of activism against violence towards women and children, community-led campaigns in response to GBVF cases reported in the media as well as NGO-led initiatives to support GBV survivors. However, these interventions were stifled by the social and cultural stigma against reporting GBVF, a lack of sustainability, decreased visibility, poor organisation and management as well as a lack of funding. Therefore, our findings show that while the South African government has made strides in its efforts to eliminate GBVF, there are no sustainable community level programming and interventions aimed at changing social norms and toxic masculinity that perpetuate GBVF. In conclusion, we recommend that efforts be made to implement intervention initiatives that go beyond creating awareness on GBVF, but partner with local NGO-led organizations to engage in programming and intervention that is aimed at changing social norms.

## 1 Introduction

Globally, 736 million (∼30%) women are said to experience violence at one point in their lives (1, 3). Worldwide the Covid-restrictions led to a proliferation of Gender Based Violence (GBV) with increases of 25% and above in certain countries. As a result, the United Nations renamed it the “shadow pandemic” (1, 3, 4). Studies show that former or current intimate partners are responsible for inflicting 87% of this violence thus leaving women unsafe in their own homes and around the people they trust (1, 3). Enaifoghe et al. (5) linked this to the violent culture that is being practiced in different social settings while Convention on the Elimination of All Forms of Discrimination Against Women (CEDAW) sees it as a social mechanism used to dominate the social, economic, and political lives of women. Women are coerced and silenced into victimisation because they fear that their partner's violent outrage will inflict bodily harm (6).

South Africa continues to have alarming levels of GBVF. These harrowing rates were further worsened during the Covid-19 national lockdown as in South Africa the 2020 crime statistics reported that 20% of women were victims of GBVF nationwide reflecting global trends of rise in GBVF during the past few years. Femicide is also prevalent across Africa—at a high rate of 3.1 per 100,000 female population, while this rate is lowest in Europe—at 0.7 per 100,000 female population (7). South Africa alone has national femicide levels that are almost five times the world's average (8, 9). This was further evidenced by South African Police Service (SAPS) revealing that between 2015 and 2020, an average of seven women's murders were reported daily (10).

In response to these concerning statistics on GBVF, the South African government has adopted certain international, regional, and local policies and ratified several human rights instruments. Strides have also been made to develop South Africa's own legislative framework to eradicate GBVF. For instance, the framework of the Sustainable Development Goals (SDGs) offers the opportunity for nations to understand and improve all action in addressing inequalities and promoting inclusive and sustainable wellbeing and development for all, particularly for those experiencing GBV and the denial of their human rights (11). It also accommodates international policy to end GBVF, with emphasis on eliminating all forms of violence against women and girls (SDG target 5.2). The 25th anniversary of the Beijing Platform for Action on Women (held in 2020) has resulted in renewed commitments from numerous governments, civil society, United Nations agencies and donors on addressing GBV against women and girls.

Locally, the South African National Strategic Plan (NSP) on GBVF (2020–2030), also known as the Emergency Response Action Plan, is an arm that was developed in response to the high levels of GBVF while the Domestic Violence Act (No. 116 of 1998), enables victims and survivors of domestic violence to apply for an interim protection order and warrant of arrest against the perpetrators of domestic violence. Thuthuzela Care Centres (TCCs) are recognised for their interdisciplinary efforts with state structures (such as the National Prosecuting Authority—*NPA*, the Department of Justice and Constitutional Development—*DOJCD*, the Department of Health—*DOH*, the Department of Social Development—*DSD* and the South African Police Service—*SAPS*) to provide professional medical care, counselling and access to investigators and prosecutors to avoid secondary victimisation, improve the process of reporting and prosecution of sexual offences (12, 13). The annual 16 Days of Activism for No Violence against Women and Children campaign has gained significance through television, radio and social media broadcasts despite its focus being primarily “women” and rarely the LGBTQI+ community (14, 15). Nevertheless, its downfall has been that during the dedicated grace period (of 16 days of activism) South Africa continues to experience exorbitant cases of GBVF.

Previous attempts to understand GBVF in South Africa have consistently excluded local communities and activists as key stakeholders (16). Chavula et al. (17) state that greater community engagement is required to address GBVF in communities, especially to be tailored correctly for pandemics. However, community members may play a significant role in combating the scourge of GBVF because of their ability to contextualise GBVF within the cultural beliefs and power struggles that organise their communities. It is important to conduct research on activists and acknowledge their role in spotlighting GBVF in their communities for government intervention. Activist movements such as *#Am I next? One in Nine Campaign* or the national *Silent Protest*, painted the extent of women's outcry and concerns for their safety in South Africa regardless of the age group (18). On the other hand, gender activists and supporters of *#SandtonShutDown* underscored GBV related matters such as pay disparities between men and women and other forms of discriminations that continue to be normalised within different structural platforms (19, 20). These movements confirm that positive outcomes are yielded when community members become a part of the government's strategic planning and GBVF solutions. Nevertheless, these mentioned examples are not research-related, but rather activist campaigns to attempt to decrease GBVF. Therefore, this study acknowledges the important role that activists and communities can play in understanding GBVF that is a complex phenomenon in itself. The better we understand this phenomenon, the easier it will be to create interventions that are sustainable and useful.

Local communities can also play a pivotal role in assisting the South African government to establish the social challenges that surround GBVF and for creating sustainable and effective solutions (21). This strategy works and was shown in crime statistics where more than 1 million serious crimes were reported by community members in 2021 (22). These serious crimes included house break ins, murder, corruption, human trafficking, and others. Again the power of communities is seen in decreasing crime levels, but has not been effectively utilized to understand the complex nature of GBVF. Social impact solutions developed without data from relevant civil society stakeholders and local communities remain ineffective in addressing the root cause of social ills. Additionally, in many settings community strategies and methods to assuage GBVF are already in place and have proven to work effectively. Many of these solutions have been community-initiated and not commenced after the incorporation of research findings. Such systems need to be acknowledged, assessed, and where required, strengthened. To this end, this study aimed to establish the known interventions to decrease or eliminate GBVF and their effectiveness from community members and activists in seven communities across South Africa.

To determine GBVF interventions knowledge and effectiveness from community members and activists, the study utilized the rights based approach (RBA). This is a framework that ensures that all citizens are protected from all forms of abuse and humiliation. Ife (23) states that human rights are important as a principle of humanity because they enable citizens to maximize their self-determination, as well as exercise their freedom and liberties. The democratic process in South Africa has ensured that the legal framework of South Africa looks different from its previous government. Miller and Redhead (24) highlight that RBAs have been essential in ensuring that the principles of non-discrimination and equality are built on participatory processes. This process will involve activist work that defends the rights of vulnerable communities through advocating for their rights. This compliments the South African legal frameworks which already includes work that has been done by activists, communities, NGOs, and the private sector. Furthermore, the legislative framework is also aligned with international instruments, policies, laws, and humanitarian agencies (23–25). However, it is the South African policies we hope to speak to since they are obliged to uphold democratic rights through the Bill of rights (26). Policies are informed by the principles of defending human rights and therefore, play an important role in eliminating the scourge of GBVF. Although South Africa has sufficient legislative frameworks to create efficient interventions and gender-responsive policies, communities and activists ultimately define and expand the definitions of freedoms. This is one of the central features of the RBA hence it has been selected as a suitable approach for this study.

## 2 Methodology

The research utilised a mixed methods design that included qualitative and quantitative components. The study included one hundred and ninety-one (191) participants in a survey for the quantitative and qualitative aspects of the study. The findings presented here were from seven communities across South Africa, namely Evaton in Gauteng; Rooibok Village in Acornhoek Bushbuckridge, Mpumalanga; Humulani Village in Phalaborwa Limpopo; Vredefort in Free State; Makgogwane Village in Ramatlabama Mafikeng in the North-West; Graslaagte in Humansdorp in the Eastern Cape and Tongaat Kwa-Zulu Natal. Data was triangulated across both qualitative and quantitative methods to promote validity in the study. The qualitative aspect of the study collected data using focus group discussions, one for male community members, another for female community members and the final one for male and female activists. Data was transcribed, coded and analysed through thematic analysis.

The quantitative component of the research consisted of a survey that collected primary data through a questionnaire. This data was collected and captured by data capturers before analysis was conducted in STATA 15. Quantitative data analysis entailed describing the entire study sample looking at the demographic, socio-economic, and intervention factors worth capturing in the survey. The study also investigated the bivariate relationship between background factors and interventions. These bivariate links were tested with the Chi-squared test at a significance level of 5% if more than 80% of the frequencies in the cross tabulations were above five, otherwise the fisher's exact test was implemented at the same significance level.

## 3 Results

### 3.1 Description of participants

The study sample included 191 participants while the response rate was estimated at 85%. The age of participants ranged from 18 years- to 76 years old while the median age was 39 years. Participants were 80% Black, 10% Coloured and 10% Indian. The sample consisted of 40% males and 60% females. With regards to ethnicity the participants were predominantly Sotho at 65%, followed by Zulus at 16%, Xhosas at 11%, Tswana at 5% and Shona at 3%. Approximately 49% were from rural areas, 34% from urban areas while 17% lived in informal settlements. The sample consisted mainly of Christians (86%) followed by individuals that were traditional at 7% and other religious beliefs made up 4%. The education attainment of participants comprised of 54% with secondary education, 39% with tertiary level education and 5% with primary education. Approximately, 70% of the sample was unemployed while 30% were working. The participants' income sources included the child grant for 44%, self employment for 21%, wages for 22% and pension grants for 13%.

### 3.2 Research site

This study was conducted in seven communities across South Africa, namely Evaton in Gauteng; Rooibok Village in Acornhoek Bushbuckridge, Mpumalanga; Humulani Village in Phalaborwa Limpopo; Vredefort in Free State; Makgogwane Village in Ramatlabama Mafikeng in the North-West; Graslaagte in Humansdorp in the Eastern Cape and Tongaat Kwa-Zulu Natal. Evaton is a small town in Johannesburg that is found in the Emfuleni local municipality, it houses 605,504 people as of 2016. The working age population forms 69.5% of the total population, with the unemployment rate sitting at 34.7%. 38.7% homes are headed by females. Bushbuckridge Local Municipality consists of 135 settlements and is divided into 34 wards, with a population of 750,821. Majority of the population is people between ages of 15 and 65 at 61.9%. The most spoken language is Tsonga at 57%, followed by Northern Sotho at 25%. Ba-Phalaborwa local municipality is a category B found in the Mopani Districts. It has 5 traditional authorities named Makhushane, Majeje, Mahishimale, Maseke and Seloane. The greatest percent of the population is made up of people of the working age, aged 15–64. There are 188,603 people as of with unemployment rate sitting at 37.8%. Vredefort, also known as Mokwallo, is a farming town in the Free State. It houses 14,619 people, where 46.77% are males and 53.27% are females. Ramatlabama is a village in the Mafikeng local municipality, located at the Botswana border. It is made up of 6 smaller villages. It has a population of 2,046 as of 2016. Humansdorp is a town in the Kouga Local Municipality which houses 21,894 people. Males are 48.35% and females are 51.56%. It is mostly made up of Coloured nationals. Finally, Tongaat is a small town that falls within the eThekwini local municipality, it houses 42,554 people as of 2016. Majority of the population is Indian/Asian nationals.

### 3.3 Qualitative findings

#### 3.3.1 Awareness campaigns on GBV and violence

Participants reported being aware of a number of awareness campaigns on gender-based violence and femicide in the communities. Some of the awareness campaigns were spearheaded by community-based organization resources constrained. Participants were also aware of such awareness programme at national level such as the 16 Days of activism government-initiated programmes and through mainstream media messages on combating. There is a broad awareness of the 16 Days of violence against worm and children in South African and there has been huge criticism from activists about the impact of the campaign. Gives solidarity and support to women and children around the world through creating awareness about GBV and empowering to fight back. The main criticism in South Africa is that it is limited to awareness and with no concrete intervention plans to bring services that are needed in communities to combat violence. What participants are stating belong is that while the awareness is needed there is more that is needed for deal with GBV. The lack of services to deal with GBV has trapped many women in the cycle of violence.

*Activist1: I'm involved in different aspects. Like one, I work in a drop-in center, but we have different programs. In the drop-in center, we have awareness campaigns on GBV, violence and others.*

*A4: We have tried to do community outreach programs as Thuthuzela Centre, we have noticed this every 3 months when I'm doing the statistics*.

*A1: That is why In many cases to fix this thing, even if we go to social workers to seek help even if they give us programs. It's hard for us to heal, that is why the cycle continues and we still have such GBV cases*.

*A2: To just to respond to you, prevention is better than cure. There are awareness campaigns happening in our communities, but once this person has violated another, let us take him to relevant authorities and not say that we will take both of them, because this case will not end up going to the correct places*.

### 3.4 Community members as policing forum patrollers

Participants in this study reported that there are community led interventions. This was in the form of Community Policing Forum patrollers who try to ensure the safety, order and peace in communities while dealing with different forms of crime.

*A1: You see my sister when it comes to patrolling the street….. So, what we can do, we only take those things, and it's either we call the police, or we let them go, we will then decide, because we have old people that we work with, Our job is to run and catch the thieves. Then when it comes to GBV when we go when we are called or on suspicion. But it's not in many cases, when a woman will come and say, my partner is beating me*.

#### 3.4.1 Safe houses

Safe houses also known as shelters were cited as one of the intervention available as an intervention available in the community as a temporary intervention to women experiencing GBV.

*A3: They will take me to a safe house now, but it's going to be only for three months, but when I come out of it, I'll start life afresh. They do give them skills but remember they cannot stay there forever. It's only for two months after three months, you need to exit. Yes, it becomes an issue that three months is small. One does not look big look beyond. Most people do not look beyond that when I arrive at safe houses, I must do this and that and when I go out, I will do this and that they just go because they're already fed up*.

*A4: and whilst this lady has gone to a place of safety. She will also be receiving services from that social worker that will be intervening on the case a case.*

*A5: even if they can be taken to a safe place and stay there for a certain period of time and come out after three months. But when they come back to the community where will they go? They will go back to their partners*.

#### 3.4.2 Social worker intervention

The other form of intervention reported in this study was the professional social worker intervention rendered in the form of psycho-social services to individuals who experienced GBV and their families. In the South African context social workers take a stance of supporting the development and empowerment of women through encouraging the victim to take an active part in shaping their future as a survivor of abuse and violence. Often the type of intervention provided by social workers was providing the client with adequate information while respecting the woman's right to self-determination. Social workers in community organisations also engaged in a screening process of clients to assess the family environment that victims were from, type of violence experienced by the woman and her children and the social circumstances present. In response, an appropriate plan for intervention and disposition of the case would follow such as divorce or reconciliation through mediation. However, participants alluded that although family intervention was important, it was inadequate due to being unable to address the root causes of the violence taking place. Consequently, violence often continued in the relationship until a woman made the decision to leave.

*A5: Maybe they will stay with their family for that time because a social worker has done the intervention with the family, maybe they will come back for a month or two which is too long. They will go back to their Partners, they will tell you I was not able to stay there because of one, two, three*.

#### 3.4.3 Community taking action for themselves

Community interventions to deal with cases of GBV were also reported as one of the forms of interventions by participants in this study. This form of intervention entailed community members getting directly involved in dealing with the perpetrators when incidences of GBV occurred between couples in the community. These interventions varied between capturing the perpetrator and handing him over to the police to community members taking the law into their own hands and punishing the perpetrator as they see fit.

*YouthFem1: And this thing of intervening is difficult because you get caught up in the fight*.

*YouthF2: Yes, they end up beating you also, because there was a time, I intervened in my neighbours fight, her husband ended clapping me. We also beat up a man who beat his partner and she was in ICU because he had stabbed her. We beat him up as a community. The time the woman woke up in ICU, She said, why did we beat her husband? Who said you must beat my husband? Like she turned on us, people who were helping here so we don't help people.*

### 3.5 Quantitative results

The survey questionnaire revealed that approximately 51% of participants believed that the current GBVF intervention programmes were adequate. However, the survey revealed that only about a third (35%) of participants knew of GBVF intervention programmes that were present. The interventions known by participants included awareness campaigns offered through the Emthonjeni-Levar Mbatha Clinic, GBVF forearms programme as well as child protection forum workshops. Some participants were aware of POWA, Ke Moya, Phala and other NGOs that GBVF incidents could be reported to for obtaining assistance. Finally, two participants mentioned previous organisation of a march against GBVF, while one reported that door-to-door counselling had been offered at the community level.

Quantitative results helped us understand the levels of knowledge of GBV interventions as well as the perception of GBV interventions being adequate across various groups.

GBVF interventions were considered to be at optimum levels regardless of background characteristics, except for place of residence as no other specific groups showed statistically significant differences to others. Please refer to Table 1. The median age of individuals that considered interventions were inadequate was 40 years with an interquartile age of 17 years, while the group that considered the interventions adequate had a median age of 34 years and interquartile range of 17 years. Similar proportions of each variable category thought that interventions were adequate and inadequate with highest representation being seen among females, secondary school holders, unemployed individuals and those that had witnessed, but not experienced GBVF.

**Table 1:** Levels of GBVF intervention adequacy by background characteristics, South Africa, 2023.

Characteristic	GBVF interventions inadequate *n* = 72	GBVF interventions adequate *n* = 61	*P*-value
Age (med;IQR)	40; 17	34;17	0.043
Gender			0.697
Males	18 (25%)	24 (39.34%)	
Females	54 (75%)	37 (60.66%)	
Educational attainment			0.407
Primary	9 (12.68%)	8 (13.11%)	
Secondary	32 (45.07%)	35 (57.38%)	
University degree	30 (42.25%)	18 (29.51%)	
Employment status			0.839
Unemployed	46 (63.89%)	40 (65.57%)	
Employed	26 (36.11%)	21 (34.43%)	
Ever witnessed GBVF			0.344
No	22 (31.43%)	24 (39.34%)	
Yes	48 (68.57%)	37 (60.66%)	
Ever experienced GBVF			0.615
No	39 (54.93%)	35 (59.32%)	
Yes	32 (45.07%)	24 (40.68%)	
Place of residence			0.069
Informal settlement	16 (22.22%)	10 (16.39%)	
Rural	10 (13.89%)	15 (24.59%)	
Urban	46 (63.89%)	36 (42.62%)	

A borderline significant difference was shown by place of residence though. Individuals that considered GBVF interventions to be inadequate were predominantly from urban areas at 64% followed by those from informal settlements at 22.22%, while persons that considered interventions adequate were mainly from urban areas at 43% followed by rural areas at 25% each.

Table 2 depicts levels of GBV intervention knowledge. Our results show that this differed statitistically by education level and employment status. Individuals with higher levels of education had greater knowledge of GBVF interventions. However, this educational difference was not protective to the witnessing and experience of GBV. In other words, people with higher levels of education knew more GBV interventions yet this did not protect them from being victims of violence. Levels of knowledge of GBVF interventions were highest amongst unemployed individuals.

**Table 2:** GBVF intervention knowledge by background characteristics, South Africa, 2023.

Characteristic	No knowledge of GBVF interventions *n* = 126	Knowledge of GBVF interventions *n* = 61	*P*-value
Age (med; IQR)	41; 22	36; 17	0.227
Gender			0.182
Males	40 (31.74%)	33 (54.09%)	
Females	86 (68.26%)	28 (45.91%)	
Educational attainment			0.037
Primary	22 (17.46%)	6 (9.84%)	
Secondary	72 (57.14%)	30 (49.18%)	
University degree	36 (28.57%)	25 (40.98%)	
Employment status			0.000
Unemployed	101 (78.91%)	31 (50.82%)	
Employed	27 (21.09%)	30 (49.18%)	
Ever witnessed GBVF			0.788
No	48 (38.10%)	22 (36.07%)	
Yes	78 (61.90%)	39 (63.93%)	
Ever experienced GBVF			0.412
No	72 (58.06%)	31 (51.67%)	
Yes	52 (41.94%)	29 (48.33%)	
Place of residence			0.164
Informal settlement	21 (16.41%)	12 (19.67%)	
Rural	69 (53.91%)	24 (39.34%)	
Urban	38 (29.69%)	25 (40.98%)	

## 4 Discussion and conclusion

Qualitative findings revealed that participants were aware of a number of awareness campaigns on gender-based violence and femicide. There has been huge criticism from activists about the effect of the 16 Days campaign that gives solidarity and support to women and children around the world through creating awareness about GBV and empowering individuals to fight back. The main criticism in South Africa is that it is limited to awareness with no concrete intervention plans to bring services that are needed in communities to combat violence. Participants stated that while awareness was needed, more had to be done to deal with GBV. Subsequently, the lack of services to combat GBV had trapped many women in a cycle of violence.

Anti-GBV programming in South Africa has been rather sparse over time. Burris (27) highlights that limited funding influences organisational responses to reach fewer communities. Also, intervention efforts focus on offering services to survivors of GBV such as programmes of victim empowerment, temporary safety shelters, trauma clinics, support groups and counselling with less systemic, focus on batterer intervention (28, 29). Burris (27) argues that government's emphasis should be on ensuring major reform of the criminal justice system rather than focusing on creating adaptations within the existing system. Additionally, it is essential that systemic inequalities that contribute to GBV should be addressed urgently.

Our findings showed that community members as policing forum patrollers were more effective than the police force in communities. Historically, communities in South Africa have always had the agency to self-organize and mobilise at grassroots level to deal with the challenges that they were currently facing. These volunteers have traditionally formed platforms known as community policing forum (CPF) street patrollers. CPF patrollers work with the local police station and law enforcement to alert the police of any crime committed. The CPF was implemented in the mid 1990's in South Africa and enacted through the South African Police Services Act of 68 of 1995 (30). These initiatives have succeeded in building trust between citizens and the police since there was a history of suspicion between the police and citizens that developed during apartheid in South Africa (31, 32).

While this is one of the interventions that community members are aware of; the narrative by participants indicates that most reported cases that CPF dealt with were more general crime related incidents as few women reported GBV cases to the CPF. This alludes to GBV still being considered a very private matter in the South African context. Indeed, across the continent GBV is still considered a private matter as scholars have shown this to be the case in Tunisia, Sierra Leone and even Nigeria (33–35).

Regarding the findings on safe houses being a form of GBVF intervention according to the Department of Social Development's Minimum Standards on Shelters for Abused Women a “shelter is a residential facility providing short-term intervention for women and children in crises. This intervention includes meeting basic needs as well as providing support, counseling and skills development” (36). There is a widely recognised lack of adequate government funding to help overwhelmed NGOs provide direct support to GBV victims, including shelters. Such inconsistent government support for shelters has meant that where shelters did exist, they could not accommodate large numbers of women and were at times forced to close leaving many women without the essential services.

Also, GBV safe houses were allowed to provide refuge for a maximum of only three months. This led to GBV survivors being forced to return to the homes that they shared with their perpetrators. Numerous scholars have written about the inadequately short periods of refuge offered to GBV survivors at safe houses in South Africa and this was particularly felt during the Covid-19 pandemic (37). This is linked to lack of funding in the midst of high demand for shelter services (38). Although government believes that it is doing its best, it is essential that more be done to solve this dilemma for the survival or GBV victims.

The research highlighted the GBVF intervention role played by social workers. Leburu-Masigo and Kgadima (39) argue that although social workers are supposed to be at the forefront of all forms of crises, including pandemics and related matters of violence, the South African government did not even include them in the list of essential workers during the Covid-19 lockdown. Luvo and Saunders (40) advocate for a multi-disciplinary team approach in handling GBV to ensure that social workers can assist survivors more comprehensively in their psycho-social needs. However, such an approach would need the context to be taken into consideration.

The study showed that at times communities take matters into their own hands. These acts are also known as mob justice or “vigilante” behavior in South African Townships. They adopt the same tactics to the police in the arrest, detention, punishment of crime suspects (41). In some cases, perpetrators are handed over to police or dropped off outside police stations. This often happens after roughing up the perpetrator and taking them to the Police station with the intention to demand further punishment from law enforcement agents. What is clear in the findings is that in cases of GBV it is complex to intervene and support a woman experiencing GBV in the community because it puts community members in a difficult position particularly when the woman experiencing violence ends up protecting the abusive partner. Previous studies have illustrated the desperate attempt of communities to take matters into their own hands due to the high levels of GBV. Kabongo (42) highlights the inter-generational culture of violence within South African local communities that complicates the building of peaceful neighbourhoods. Nevertheless, despite the negative effects of mob justice it is a classic example of how grassroots community members organize themselves to ensure social cohesion and order (43).

Our quantitative results showed that there were comparable results across the groups of individuals who thought that interventions were inadequate and adequate. This may be a reflection of the dearth of anti-GBV programming that grips South Africa nationally. Due to the lack of anti-GBV efforts, people have slipped into a state of accepting the status quo. When individuals have no model to aspire to, their current state of dysfunction becomes normalized. In a country where violence has been so deeply engrained in the minds and hearts of the ordinary man on the street, it is not surprising that South Africans feel that enough is being done. Gordan (44) reiterates Gqola's (45) argument that violence is tolerated across the country and characterised by a culture of constant dread and brutality where human life is no longer sacred. Gqola (45) further argues that GBV in South Africa is omnipresent, commonplace and normalised through the dominant public discourse. Additionally, violence against women in South Africa is embedded in justificatory narratives in apartheid discourse (46–49). Boonzaier and De la Rey (50) declare that violence is linked to broader socio-cultural mechanisms that construct woman abuse as a serious social problem in South Africa. Vetten (49) argues that the “militarisation and conflict of the Apartheid era” are engrained in the country's psyche and set the context for how men relate to women from an early age. Violence against women is enmeshed in patterns of hegemonic masculinity patriarchy and oppression, which were synonymous with colonialism and apartheid (45, 46, 51). Also, Gordan (44) highlights that dissemination of discourses of subordinate femininity and feminine transgression contribute to the prevalence of violence against women in society because these discourses position men in a hierarchal corrective relationship to all women, and construct the violence perpetrated against women as a natural response to their transgression During the apartheid era legislation, racist discourse and violence were used to humiliate and remind the “non-white” population of their subordinate position in society. Similarly men use gender-based violence and the fear of such violence to shame women and keep them within specific boundaries and categories (47, 48).

The study found that there was a borderline significant difference in intervention adequacy by place of residence. This difference may also be a reflection of a combination of socio-economic status and safety of neighbourhoods that differs greatly by place of residence in the South African context. For example, individuals from communities with lower average educational levels or incomes as well as higher unemployment and poverty rates were shown in Beyer, Wallis and Hamberg (52) to have a higher likelihood of exposure to violence thus normalizing over time. Our results tally with Edwards' (53) systematic review of literature conducted globally that perceptions related to violence differ by locality, with individuals from rural areas expecting less support and interventions from government. However, Mcilwaine (54) argues that although resource availability makes GBV consequences easier to manage in urban areas, individuals living in areas with poverty, high unemployment and poor living conditions, regardless of place of residence, are more predisposed, especially in urban areas where social relations are fragmented.

Finally, our results show that people with higher levels of education knew more GBV interventions yet this did not protect them from being victims of violence. To support this finding, similar insignificant results of intervention adequacy and knowledge were seen amongst those who had experienced violence as well as those who had not. This is largely contrary to previous literature that has depicted education as a protective factor against gender-based violence in Kenya, Ethiopia as well as the Philippines (55–57). Nevertheless, literature does indicate that women from all walks of life experience GBV regardless of race and socio-economic status (58).

Despite numerous measures that have been put in place to prevent GBVF, its levels remain high nationally. This shows that the national interventions that have been implemented in South Africa to fight against GBVF need reviewing. A continued need to understand GBVF and what drives it in South Africa is necessary. Also, the call by United Nations Agenda 2030 and the SDG to *leave no one behind* invites researchers, practitioners, and policymakers to develop a more nuanced approach to doing research, including research on GBVF. Understanding GBVF in South Africa requires a deeper knowledge of the context in which violence unfolds and an insight on what people experience. This study has attempted to begin this process through asking community member and activists to highlight the known GBVF interventions as well as by determining the levels of GBVF intervention adequacy as well as knowledge of GBVF interventions by background factors.

However, the research was riddled with some limitations. The greatest of these is that the findings are from select communities across the country. Therefore our results are good representations fro the communities that were included, but not necessarily generalisable to all parts of South Africa. Nevertheless, this limitation was largely compensated for by the large sample of approximately 191 participants. Also the study was the first step in a larger project where more communities will be incorporated to determine these and other aspects of GBVF in our quest to understand this phenomenon better. Another limitation of the study is the normalized nature of violence in South Africa. This is because when certain human behavior is normalized over time, such as violence, people may no longer view it to be something to be angry about or even notice despite its terrible consequences. This acceptance of GBV is one of the reasons for under-reporting and perpetuates the culture of silence driving GBV further. This may be the reason why questions on GBVF intervention adequacy and knowledge were answered so poorly.

Notwithstanding these limitations the study does give some recommendations that will be elaborated on further in this section. Firstly, regarding practice our results show that people's level of knowledge of the existing GBVF intereventions is generally low-in other words people do not know about the present solutions that already exist. This means that they are either not happening in certain communities as much as stakeholders would like to believe or that they are not advertised well enough for a large quantity of community members to become more aware of them.

Additionally, the already available solutions that decrease GBVF indirectly or directly need to be explained and made more apparent to community members. It is not reasonable to expect people to understand indirect pathways of preventing GBVF because these are not obvious links. For example education promotion could prevent GBVF through teaching children conflict management and communication skills in the school environment. However, this needs people to understand the importance of education beyond its ability to increase ones chances of entering tertiary education as well as secure formal employment, but rather the acquisition of secondary education is a means of socializing individuals differently through teaching conflict resolution and communication in schools in non-violent ways.

We also believe that more interventions need to be brought to the community-level. According to Dartnall and Gevers (59) violence prevention is grouped into three categories: primary, secondary and tertiary. Simply put, primary prevention of violence includes programmes that aim to prevent violence before someone is harmed, while secondary and tertiary prevention are those programmes that intervene early, or follow after violence has occurred, aiming to prevent its recurrence. Primary prevention programmes usually engage with all people, whereas secondary and tertiary prevention programmes work with high-risk groups, victim- survivors or perpetrators. In the South African context there is a focus on secondary and tertiary intervention programmes. This has its benefits, but due to the high levels of violence in our society it is important to also change the attitudes and beliefs of individuals as early as possible for them to make different non-violent choices later on in life despite their exposure to violence. This would need the creation of intervention programmes geared at children as well as their parents to support the raising of children in the South African context. This stance has been supported by studies conducted by Skeen et al. (60), Gould and Ward (61) as well as Phyfer and Wakefield (62). Additionally, the other areas identified to make the most impact if targeted for primary prevention interventions include developing relationship-building skills, building gender equality and challenging hegemonic masculinities, reducing substance abuse, improved gun control as well as challenging the widespread acceptance of violence according to Gevers et al. (63).

It is important to note that communities no longer trust government officials and there are large levels of apathy. While the Constitution of the Republic of South Africa encourages participation of local communities in partnership with government at local and provincial level as part of their commitment to democracy; there are a number of barriers that have contributed to erosion of trust between government and community members (26, 64). The lack of evident service delivery in many communities and lack of confidence in leadership that community members have elected is one of the major challenges confronting communities. This leads to lack of action and participation on the part of residents as there is limited consultation in many of the government programmes that are implemented (65). When the voices of local community member are excluded in the decision making of their own development there is often limited understanding of the value of participation. Finally, there is evident lack of trust broadly in government and local leadership and this subsequently leads to apathy and in some cases of social civil unrest and protest in communities.

This research has shown us the value of including community members and activists to investigate GBVF in the South African context. It has taught us that it is important to understand the beliefs and values of individuals when conducting research in order to establish better rapport when navigating difficult research topics. Additionally, our use of a decolonial approach, going to the grassroots levels to get current contextual information as well as asking community members about GBVF applications and tech use to prevent and address GBVF in South Africa all assisted us to get richer data.

We would recommend that these different approaches be adopted by other researchers going forward. If the local community values are unknown the use of other entities already working with these communities is incredibly useful and a means to obtain access to hard to reach communities. All in all it is important to remember that the research process is a joint knowledge production process needing researchers to partner amicably with communities and other stakeholders involved. This requires researchers to remain patient, humble and receivers of knowledge as much as they may think of themselves as suppliers of knowledge.

Finally regarding policies it is important that the National Strategic Plan (NSP) on GBVF is simplified and localized by government. The South African NSP on GBVF (2020–2030) was developed in response to the national high levels of GBVF. One of its aims is to establish the national economic cost of GBVF and ascertain that advocacy and prevention efforts have multisectoral funding models and therefore, work towards effective national responsive mechanisms in tackling GBVF. As a multisectoral plan, it seeks to respond to all violence against women [across age, location, disability, sexual orientation, and other diversities] (66). It is founded on 6 pillars namely Accountability, Coordination & Leadership; Prevention and Rebuilding Social Cohesion; Justice Safety & Protection; Response Care Support and Healing; Economic Power as well as Research and Information Management Systems (66). Government and other stakeholders have prioritised these pillars to ensure that this policy shows accountability, responsiveness, and prevents GBVF. The policy has been praised for its collective and multisectorial inputs that ensure that GBVF is addressed urgently and strategically. However, we hope that this study will assist policy makers to adopt this and other GBVF-related policies to ensure the combatting of GBVF within South African communities.

## Data Availability

The datasets presented in this article are not readily available because of ethical clearance, the dataset will be kept solely by the authors. Requests to access the datasets should be directed to motlalepule.nathane-taulela@wits.ac.za.
